# Bad Temper and Insanity

**Published:** 1881-06

**Authors:** 


### BAD TEMPER AND INSANITY.

Passionate people, the hasty kind, who flare up in a blaze, like fire to tow, or a coal to powder, without taking time to inquire whether there is any ground for such a pyrotechnic display, and then get more furious, when they find out there was no cause for their fiery feats, may learn a useful, as well as a serious lesson from an item in Dr. Blanchard's report of the King's County Lunatic Asylum, that "*three men and three women became insane by uncontrollable temper.*"

We all feel a sympathy for one who has become demented from loss of kindred, from disappointment, and from a hard lot in life; but we can have no such feeling for quarrelsome, ill natured, fretful, fault finding, complaining, grumbling creatures, the greater part of whose every day life tends to make those whose calamity it is to be bound to them, as miserable as themselves. I consider ill nature a crime, and like other crimes, is ordained in the government of God, to meet, sooner or later, its merited reward. Other vile passions may have some points of extenuation, the pleasure for example, which may attend their indulgence, but ill nature, that is, a fretful, fault finding spirit, in its origin, action and end, has no extenuating quality; and in the application of the Scripture principle, "*with what measure ye mete, it shall be measured to you again,*" will find a pitiable end. Therefore, with all the power God hath given you, strive, reader, and strive for life, to *mortify* this *deed of the flesh.* Watch hourly, watch every moment against the indulgence of a hasty temper as being offensive to your Maker, and contemptible in the eyes of your fellow man; contemptible, because for the person who possesses it, and knows it, yet indulges in it, and makes no effective efforts to restrain it, no human being can have any abiding attachment or respect, founded as it is, in low morals, or low intellect, or both.

### HARDENING THE CONSTITUTION.

Men talk about "*hardening the constitution*," and with that view, expose themselves to summer's sun and winter's wind, to strains and over efforts, and many unnecessary hardships. To the same end, ill informed mothers souse their little infants in cold water day by day; their skin and flesh, and bodies, as steadily growing rougher and thinner, and weaker, until slow fever, or water on the brain, or consumption of the bowels, carries them to the grave; and then they administer to themselves the semi-comfort and rather questionable consolation, of its being a *mysterious dispensation* of Providence, when in fact, Providence had nothing to do with it: He works no miracle to counteract our follies.

The best way I know of *hardening the constitution*, is to take good care of it, for it is no more improved by harsh treatment, than a fine garment or new hat is made better by being banged about.

### NEW YORK AND LONDON MORTALITY.

People live longer in London than in any other large city in the world. And excepting epidemics, it is more healthy than the country, notwithstanding its dirty streets, its countless cesspools, its infecting cellars, and thousand other sources of disease.

| | | | | | |
|---|---|---|---|---|---|
| 31 | die each year | out of 1000 | under | 20 years of age. | |
| 10 | " | " | from | 20 to 40 | " |
| 23 | " | " | " | 40 to 60 | " |
| 72 | " | " | " | 60 to 80 | " |
| 224 | " | " | " | 80 upwards. | |

Estimating for the first week in July, 1873. one person a week dies in London out of seventeen hundred and ninety-six, while in a corresponding week in New York, one person dies a week, out of five hundred and eighty-five.

In London, each week, 1 dies out of every 1796.
In New York, " 1 " " 585.

According to this calculation, three times as many die in New York as in London, according to the population. Chemical analysis seems to point to the fact that smoke is a great absorbent of noxious emanations. London is enveloped in everlasting smoke. Can it be that the remarkable exemption of Pittsburgh from cholera is partially owing to this cause? It is worthy of observation.

---

AVERAGE ILLNESS AT DIFFERENT AGES. — It is stated that between the ages of 20 and 30, each person has on an average nearly 7 days' illness a year; at 40 it is increased to 8 days; at 45 to 9; at 50 to 11 1-2; at 55 to 14; at 60 to 18 3-4; at 65 to 27 1-2; at 70 to 43 1-2; and 75 to 66; and at 80 to 97 1-2.

---

### PETER'S WIFE'S MOTHER;

### OR, THE LONG-WINDED SERMON.

In a small town where the custom was to toll the village church bell when any of the citizens died, a clergyman announced for his text, "*And Peter's wife's mother was laid and sick of a fever.*" He found some means of expatiating on it largely, to the extent of several discourses. At length there was very decided murmuring among the townsfolk, ending in a deputation to the parson, requesting a change of subject, but it failed of its effect. And while the congregation were devising some more decided procedure, the village bell commenced tolling, to the surprise of every one, as it was not known that any one was sick; several persons went directly to the church and inquired with considerable anxiety who was dead? "*Peter's wife's mother*," replied the sexton.

LESSON.—*Do not spin out your discourses to the little end of nothing sharpened; let them be filled with great and weighty truths, and leave it to your hearers to expatiate them in practical life.*

### WINTER SHOES.

LIKE the gnarled oak that has withstood the storms and thunderbolts of centuries, man himself begins to die at the extremities. Keep the feet dry and warm, and we may snap our fingers in joyous triumph at disease and the doctors.

Put on two pair of thick woollen stockings, but keep this to yourself, go to some honest son of Saint Crispin and have your measure taken for a stout pair of winter boots or shoes; shoes are better for ordinary, every-day use, as they allow the ready escape of toe-odors, while they strengthen the ankles by accustoming them to depend on themselves A very slight accident is sufficient to cause a sprained ankle to an habitual boot wearer. Besides, a shoe compresses less, and hence admits of a more vigorous circulation of the blood. But wear boots when you ride or travel. Give direction, also, to have no cork or Indian rubber about the shoes, but to place between the layers of the soles, from out to out, a piece of stout hemp or tow linen which has been dipped in melted pitch. This is absolutely impervious to water—does not absorb a particle—while we know that cork does, and after a while becomes "soggy" and damp for weeks. When you put them on for the first time, with your ordinary socks, they will feel as "*easy as an old shoe*," and you may stand on damp places for hours with impunity

